# An Unusual Subcutaneous Mass in an Otherwise Healthy Patient: A Case Report of Localized Histoplasmosis Diagnosed on Excisional Biopsy

**DOI:** 10.1155/2017/9485793

**Published:** 2017-10-04

**Authors:** Brendon M. Esquibel, Christine J. Waller, William A. Agger

**Affiliations:** ^1^Department of Medical Education, Gundersen Medical Foundation, La Crosse, WI, USA; ^2^Department of General Surgery, Gundersen Health System, La Crosse, WI, USA; ^3^Infectious Disease Section, Gundersen Health System, La Crosse, WI, USA

## Abstract

Patients are commonly referred to general surgery clinics for evaluation of soft-tissue masses of the trunk and extremities. The primary goal of surgical referral is to confirm the presence of a mass, to assess the need for additional imaging and/or testing, and to gauge amenability to surgical biopsy, whether incisional or excisional. This is a case of a 67-year-old woman who was referred to surgery clinic for a small soft-tissue mass near her left elbow that had increased in size and pain over the past year. The mass had been present for several years. After MRI imaging revealed a nonspecific process, an excisional biopsy was performed. Following a careful review of the patient's history, risk factors, and histological results, a diagnosis of localized subcutaneous* Histoplasma capsulatum *var.* capsulatum* infection was made. Without signs or symptoms of active, systemic disease, no further treatment was recommended. The patient was provided risk factor counseling for symptoms or signs of active histoplasmosis and outpatient follow-up. Histologically, most masses will return as benign and mesenchymal in origin. However, soft-tissue masses may arise from uncommon etiologies and a broad differential is needed to ensure appropriate management and recommendations.

## 1. Introduction

Patients are commonly referred to general surgery clinics for evaluation of soft-tissue masses of the trunk and extremities. These lesions can range from common, benign masses of multiple etiologies to rare, malignant tumors that can be life-threatening. The primary goal of surgical referral is to confirm the presence of a mass, to assess the need for additional imaging and/or testing, and to gauge amenability to surgical biopsy, whether incisional or excisional.

Histologically, most masses will return as benign and mesenchymal in origin, with lipomas being the most common histologic type. However, soft-tissue masses may arise from uncommon etiologies and a broad differential is needed to ensure appropriate management and recommendations. We present a case of 67-year-old woman who presented to a tertiary care teaching hospital with a small soft-tissue mass. She had multiple extrinsic risk factors for a deep-tissue fungal infection and was ultimately diagnosed with localized subcutaneous histoplasmosis by history, physical examination, and histological results following excisional biopsy.

## 2. Case

A 67-year-old woman was referred by her primary care provider to surgery clinic for a small soft-tissue mass near her left elbow. The mass had been present for multiple years. Over the past few months it had become increasingly painful, and it had increased in size over the past year. She said that she had experienced no overlying skin changes, drainage, or known trauma to the extremity. She had no other known lesions, masses, or adenopathy.

The initial physical examination demonstrated a mobile, 2.5 cm × 2 cm soft-tissue mass over the dorsal aspect of the left forearm, just distal to the olecranon process. The mass was not tender to palpation, and there were no overlying skin changes or regional adenopathy. Results of an examination of her head, neck, chest, abdomen, and other extremities were normal. A magnetic resonance imaging (MRI) scan of the patient's elbow was obtained, which revealed a nonspecific, poorly defined infiltrative process within the subcutaneous fat, with a broad differential diagnosis including lipoma, sarcoma, nodular fasciitis, or benign versus malignant fibrous histiocytoma.

**Figure 1:**
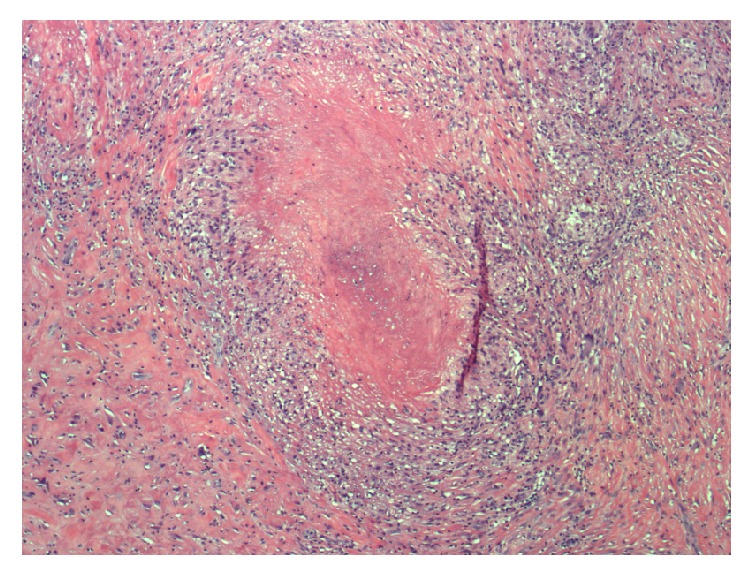
Haematoxylin and eosin stain of biopsy specimen revealed necrotizing granulomatous inflammation with small, round-oval budding yeast forms with narrow-based budding.

**Figure 2:**
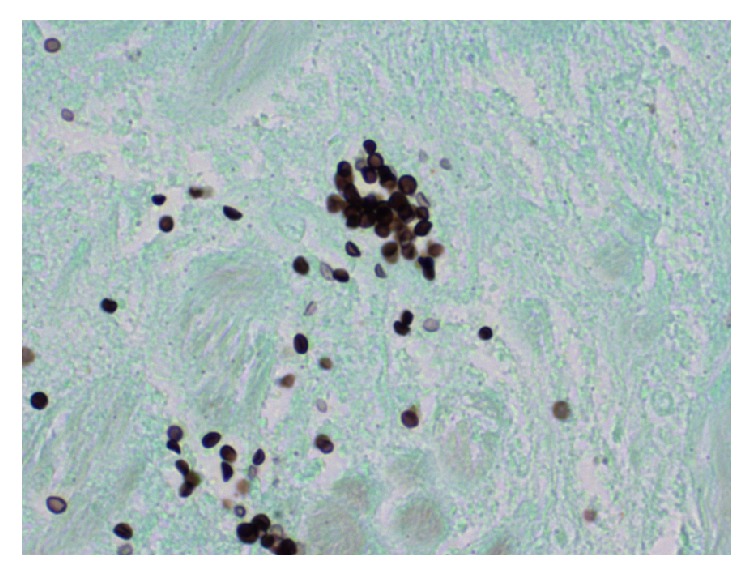
Grocott methenamine silver (GMS) stain of the same biopsy specimen as in Figure 1.

Based on these findings, surgical excisional biopsy was recommended. The biopsy was collected in formalin and the haematoxylin and eosin stain revealed necrotizing granulomatous inflammation with small, round-oval budding yeast forms with narrow-based budding (Figure 1). Further histologic findings by Grocott methenamine silver (GMS) staining were consistent with a deep fungal infection, in the likely differential diagnosis order of histoplasmosis, blastomycosis, sporotrichosis, and coccidioidomycoses. Although definitive identification could not be performed on paraffin fixed tissue, the presence of ovoid, GMS-positive yeast 2 *µ*m to 4 *µ*m in diameter with single, narrow-based buds was consistent with* Histoplasma capsulatum (H capsulatum) *(Figure 2) [1]. In the granuloma, these yeast cells were more frequent than what is commonly seen with sporotrichosis, did not reveal the broad-base budding typically seen with blastomycosis, and did not have the large spherules seen with coccidioidomycosis. Serum antigen and antibody studies were negative for histoplasmosis and blastomycosis, and a chest radiograph revealed no signs of pulmonary disease.

In addition to the patient's residence along a Mississippi River tributary, further epidemiologic history was obtained revealing multiple high-risk fungal exposures, including a history of rose gardening (too remote for sporotrichosis), frequent travels to mountainous Mexico (high altitude only, well outside of the Sonoran Life zone of coccidioidomycosis), and living in Southwestern Wisconsin (outside Wisconsin's hyperendemic area of blastomycosis). However, additional inquiry into her social history was notable for the long duration of bat colony in the attic of her home that had caused guano and urine contamination of the underlying living space.

Following a careful review of the patient's history, risk factors, and histological results, a diagnosis of subcutaneous* Histoplasma capsulatum *var.* capsulatum* (*H capsulatum*) infection was made. Without signs or symptoms of active, systemic disease, no further treatment was recommended. The patient was provided risk factor counseling for symptoms or signs of active histoplasmosis and monitored in the Infectious Disease clinic, and remained healthy over the subsequent 18 months.

## 3. Discussion

The clinical differential diagnosis for this patient's soft-tissue lesion included benign and malignant soft-tissue tumors, lymphoma, noninfectious inflammatory changes, and infection. Magnetic resonance imaging, although helpful in defining the anatomic location of the mass and excluding vital structure involvement, was unable to further refine the differential diagnosis, and excisional biopsy was completed. The patient's histologic studies and epidemiologic history, including prolonged exposure to bat guano from the bat colony in her attic and her geographic locale within the Mississippi River Valley system, supported the diagnosis of histoplasmosis. Given that the patient's chest radiographic, antibody, and urinary antigen studies were negative, this was an unusual case and likely represented a localized, subcutaneous histoplasmosis infection due to traumatic inoculation. This rare occurrence has been reported in the past [2].

Fungal infections can cause a wide range of diseases in humans. In the United States, deep-tissue mycoses are common but usually asymptomatic, and recent studies suggest an increased incidence of these infections in the Midwest, especially in elderly populations [3]. As ubiquitous and highly adaptable organisms, these fungi can propagate in almost any environment and present in human hosts in a variety of ways. The etiologies of these infections depend, in part, on epidemiological settings. As in our case, obtaining a careful epidemiologic history, including information about a patient's immune status, geographical locale, travel history, recent trauma or surgery, previous antimicrobial therapy, lifestyle/hobbies, and animal exposure or bites, is essential when developing a comprehensive differential diagnosis. A detailed workup and evaluation for systemic disease—negative in this case—is also necessary following the diagnosis of any subcutaneous fungal infection.

Endemic mycoses, such as histoplasmosis, blastomycosis, and coccidioidomycosis, are known to affect both healthy and immunocompromised individuals and tend to arise in specific geographic locations. Usually developing after a primary pulmonary infection, these fungi have the capacity to involve both the skin and deep organs. Thus, the endemic fungi can range from localized skin and soft-tissue infections to disseminated disease involving multiple organ systems. For disseminated disease, the point of entry is typically through the respiratory tract, from which the infection can spread to other sites [4]. In rare cases, the organisms gain direct access through skin, where they can propagate and cause localized infections. Such localized subcutaneous infections are uncommon and typically originate with the contamination of skin surfaces followed by the traumatic inoculation of organisms into the dermis and/or subcutaneous tissues [5].

When histoplasmosis first came to medical attention in the early 1900s, it was characterized as a rare but severe systemic infection that was inevitably fatal in most patient groups [6]. More recent epidemiologic studies have demonstrated that most histoplasmosis infections follow pulmonary infections and are subclinical and that only a small percentage will progress to severe, disseminated disease, typically in immunocompromised patients. The most common entry point for* H capsulatum* is the lung. Localized cutaneous and subcutaneous infections with* H capsulatum *are rare, with only a handful of cases described, the majority of which were in immunosuppressed patient groups [1, 7–10].

Infection may be due to direct inoculation or reactivation of preexisting, quiescent subcutaneous disease in patients with previous subacute pulmonary histoplasmosis. Although distribution of* H capsulatum* is worldwide, it is hyperendemic to eastern North America, notably the mid-to-lower Ohio and Mississippi River valleys. Bird and bat excrement enhance the growth of the organism in soil by accelerating sporulation. As in our case, environmental sites are typically visibly contaminated by heavy accumulations of bat or bird guano.

Most cases will be subclinical in nature, but a prompt and accurate diagnosis of any potential fungal infection is important to improving morbidity and mortality in some infected patients. A comprehensive history, including a detailed travel history, and a complete physical examination are needed at the time of initial evaluation. Chest radiographs and/or computed tomography scans are useful in finding the characteristic features of pulmonary disease. Isolation of the organism with histopathology and/or culture is the gold standard to confirm diagnosis and speciation, although this can be time consuming and insensitive. Various laboratory tests have been developed that may provide early clues to the diagnosis.* Histoplasma* antibody and antigen detection of fungal cell elements in urine, serum, and cerebrospinal fluid by enzyme-linked immunoassay were proven to be important in the diagnosis, treatment monitoring, and prognosis of acute disseminated histoplasmosis, but they are not sensitive tests for a localized, subcutaneous infection. Fungal cultures can also be useful in patients in whom there is concern for either local or systemic disease [1]. In cases of isolated subcutaneous histoplasmosis, lesions are typically self-limiting, and therapy is indicated only for prolonged episodes or in immunosuppressed individuals.
